# Relationship between Vitamin D and the development of atrial fibrillation after on-pump coronary artery bypass graft surgery

**DOI:** 10.5830/CVJA-2016-064

**Published:** 2017

**Authors:** Levent Cerit, Hatice Kemal, Kamil Gulsen, Barcin Ozcem, Zeynep Cerit, Hamza Duygu

**Affiliations:** Near East University, Nicosia, Cyprus; Near East University, Nicosia, Cyprus; Near East University, Nicosia, Cyprus; Near East University, Nicosia, Cyprus; Near East University, Nicosia, Cyprus; Near East University, Nicosia, Cyprus

**Keywords:** atrial fibrillation,, Vitamin D,, coronary artery bypass graft surgery

## Abstract

**Background:**

Vitamin D deficiency is associated with many diverse cardiovascular disorders, such as hypertension, heart failure, stroke, coronary artery disease and atrial fibrillation. The relationship between Vitamin D and the development of atrial fibrillation after coronary artery bypass surgery (CABG) has not been studied. Therefore, we assessed the relationship between Vitamin D and the development of postoperative atrial fibrillation (POAF) after CABG.

**Methods:**

Medical records of consecutive patients who underwent CABG surgery were retrospectively reviewed for the development of atrial fibrillation in the postoperative period. Vitamin D, other biochemical parameters, and clinical and echocardiographic parameters were evaluated in all patients. The independent variables for the development of postoperative atrial fibrillation were defined and their predictive values were measured.

**Results:**

The study group consisted of 128 patients, of whom 41 (32%) developed POAF. Age, diabetes mellitus, chronic obstructive pulmonary disease, history of transient ischaemic attack/stroke, heart failure, left atrial diameter, platelet:largecell ratio, and creatinine, urea, uric acid, calcium and potassium levels were identified as important variables for the development of POAF. However, with logistic regression analysis, chronic obstructive pulmonary disease (OR: 28.737, 95% CI: 0.836–16.118, p < 0.001), heart failure (OR: 15.430, 95% CI: 0.989–7.649, p = 0.006), diabetes mellitus (OR: 11.486, 95% CI: 0.734–11.060, p = 0.001) and left atrial diameter (OR: 1.245, 95% CI: 0.086–6.431, p = 0.011) appeared as independent variables predicting the development of POAF.

**Conclusion:**

In our study, although there was a significant negative correlation between Vitamin D and left atrial diameter, Vitamin D level was not an independent predictor for POAF.

## Background

Atrial fibrillation (AF) is the most common arrhythmia occurring after coronary artery bypass graft (CABG) surgery and is seen in approximately 15 to 30% of patients. The occurence of postoperative atrial fibrillation (POAF) is associated with increased morbidity and mortality rates, longer hospital stay and a two- to three-fold increase in incidence of postoperative stroke. Older age, obesity, hypertension (HT), prior AF and cognestive heart failure are associated with a higher risk for POAF.^1^

Vitamin D is transformed in the liver and kidneys to calcidiol and calcitriol, respectively, and affects specific target tissues via Vitamin D receptors (VDRs). Calcitriol, the active form of Vitamin D, binds to VDRs in the intestines, bones and kidneys to increase calcium absorption from the intestines, promoting calcium deposition in the bones. VDRs are found in other tissues, including the brain, cardiomyocytes, vascular smooth muscle cells, endothelial cells, pancreatic beta-cells, skeletal muscle, the prostate, colon, macrophages and skin, exerting several pleiotropic effects. Vitamin D utilises a direct effect relating to atherosclerosis, such as modulating endothelial function and influencing vascular smooth muscle proliferation and migration.^2^,^3^

To our knowledge, the relationship between Vitamin D and POAF has not been studied before. Therefore, we assessed the relationship between Vitamin D and the development of POAF.

## Methods

The study group consisted of 128 consecutive patients whounderwent on-pump CABG surgery. The data of the patients were retrospectively analysed for AF in the postoperative period until discharge. The study was approved by the local ethics committee.

The patients were monitored using a heart-rhythm monitor in the intensive care unit. In addition, daily electrocardiographic recordings were obtained during the hospital stay, both in the intensive care unit and the regular ward. New-onset postoperative AF (as classified by the Society of Thoracic Surgeons) was defined as AF or atrial flutter occurring in the postoperative period and requiring medical treatment (beta-blocker, calcium channel blocker, amiodarone, anticoagulants and cardioversion). Patients who developed AF in the postoperative period up to discharge were included in the POAF group.

Patients’ data, including age, gender, history of HT, chronic kidney disease, diabetes mellitus (DM), heart failure (HF), chronic obstructive pulmonary disease (COPD), congenital heart disease, valvular heart disease, liver disease, stroke, thyroid disease, pre-operative drug use (beta-blockers and statins), and echocardiographic variables such as ejection fraction (EF), left atrial diameter, and presence of valvular disease were retrospectively retrieved from the medical charts and included in the analysis.

All patients underwent transthoracic echocardiography using a Vivid S5 (GE Healthcare) echocardiography device and Mass S5 probe (2–4 MHz). Standard two-dimensional and colour-flow Doppler evaluations were acquired according to the guidelines of the American and European Societies of Echocardiography.^4^ The EF was measured according to Simpson’s method. Left atrial diameter was measured in parasternal long-axis view using two-dimensional echocardiography at the end-systole of left ventricular systole.

Study exclusion criteria were patients with paroxysmal or persistent AF, being on anti-arrhythmia medication, patients who underwent pharmacological or electrical cardioversion before CABG surgery due to reasons other than AF, patients who underwent other cardiac procedures in addition to CABG or who were planned to undergo emergency surgery, and patients who had significant valvular disease or prosthetic valvular disease.

Levels of 25-hydroxy (OH) Vitamin D, calcium and other biochemical and haematological parameters were measured following a fasting period of eight hours. Serum 25-(OH) Vitamin D levels were measured by chemiluminescence immunoassay using a Lıaıson analyser (DiaSorin Inc). Vitamin D deficiency was defined as serum levels of 25-(OH) Vitamin D < 20 ng/ml and Vitamin D insufficiency was defined as a level of 20–29 ng/ml. Plasma levels of 25-(OH) Vitamin D > 30 ng/ml were defined as normal.

## Statistical analysis

Statistical analysis was performed using the SPSS (version 20.0, SPSS Inc, Chicago, Illinois) software package. Continuous variables are expressed as mean ± standard deviation (mean ± SD) and categorical variables as percentage (%). The Kolmogorov–Smirnov test was used to evaluate the distribution of variables. The Student’s t-test was used to evaluate continuous variables showing a normal distribution, and the Mann–Whitney U-test was used to evaluate variables that did not show a normal distribution. A p-value < 0.05 was considered statistically significant.

## Results

This study included 128 consecutive patients, of whom 41 (32%) developed POAF. The main characteristics of patients who developed POAF and those who did not are presented in Table 1. All patients were on beta-blocker and statin therapy, and 93.7% were on angiotensin converting enzym inhibitor/angiotensin receptor blocker therapy. Comparisons of different laboratory and echocardiographic parameters are presented in Table 2.

**Table 1:** Patient characteristics

	POAF		
Patient characteristics		Present	Absent	p-value
Age (mean ± SD)		67.6 ± 8.6	63.9 ± 9.8	0.047
Body mass index (mean ± SD)		27.2 ± 3.7	26.9 ± 4.1	0.755
(median)		(25.8)	26.7	
Gender, n (%)	Male	35 (85.4)	77 (88.5)	0.616
	Female	6 (14.6)	10 (11.5)	
Hypertension, n (%)	+	40 (97.6)	77 (88.5)	0.104
	–	1 (2.4)	10 (11.5)	
Diabetes mellitus, n (%)	+	31 (75.6)	26 (29.9)	< 0.001
	–	10 (24.4)	61 (70.1)	
TIA/stroke, n (%)	+	5 (12.2)	1 (4.7)	0.013
	–	36 (87.8)	86 (98.9)	
COPD, n (%)	+	15 (36.6)	5 (5.7)	< 0.001
	–	5 (5.7)	82 (94.3)	
Heart failure, n (%)	+	12 (29.3)	3 (3.4)	< 0.001
	–	29 (70.7)	84 (96.6)	

POAF, postoperative atrial fibrillation, TIA, transient ischaemic attack, COPD, chronic obstructive pulmonary disease.

**Table 2:** Laboratory and echocardiograpic parameters

	POAF		
Laboratory and echocardiographic parameters	Present mean ± SD (median)	Absent mean ± SD (median)	p-value
Haemoglobin (g/dl)	13.5 ± 1.7 (13.1) (13.1)	13.7 ± 1.5 (13.7) (13.7)	0.316
Platelets (10^3^/μl)	218.9 ± 59.7 (212)	234.1 ± 66.6 (230)	0.68
White blood cells (10^3^ cells/μl)	7.8 ± 2.3 (7.6)	7.6 ± 2.2 (7.5)	0.647
Mean platelet volume (fl)	10.5 ± 1.1 (10.5)	10.4 ± 0.9 (10.4)	0.303
Neutrophils (10^3^ cells/μl)	4.7 ± 2.1 (4.6)	4.3 ± 1.2 (4.2)	0.384
Lymphocytes (10^3^ cells/μl)	1.9 ± 0.8 (1.9)	2.3 ± 1.5 (1.9)	0.072
Neutrophils:lympocytes	2.9 ± 2.0 (2.5)	2.1 ± 0.8 (1.9)	0.136
Platelet:large cell ratio	33.6 ± 16.2 (29.4)	27.5 ± 6.7 (27)	0.006
Sedimentation (mm/h)	27.4 ± 23.2 (23.5)	24.9 ± 20.4 (19)	0.758
Urea (mg/dl)	46.8 ± 22.2 (41)	36.7 ± 4.3 (32)	0.012
Creatinine (mg/dl)	1.07 ± 0.29 (1)	0.94 ± 0.24 (0.8)	0.013
(mmol/l)	(94.59 ± 25.64) (88.4)	(83.10 ± 21.22) (70.72)	
Fasting plasma glucose (mg/dl)	136.7 ± 52.2 (110)	120.3 ± 40.3 (106)	0.340
(mmol/l)	(7.59 ± 2.90) (6.11)	(6.68 ± 2.24) (5.88)	
C-reactive protein (mg/dl)	1.6 ± 2.5 (0.5)	0.8 ± 1.2 (0.3)	0.053
Total cholesterol (mg/dl)	179.2 ± 45.1 (178)	183.8 ± 53.3 (179)	0.680
(mmol/l)	(4.64 ± 1.17) (4.61)	(4.76 ± 1.38) (4.64)	
High-density lipoprotein cholesterol (mg/dl)	39 ± 8.3 (37)	39.3 ± 11.6 (37)	0.760
(mmol/l)	(1.01 ± 0.21) (0.96)	(1.02 ± 0.30) (0.96)	
Low-density lipoprotein cholesterol (mg/dl)	112.6 ± 38.5 (111.5)	114.9 ± 46.9 (101)	0.920
(mmol/l)	(2.92 ± 1.00) (2.89)	(2.98 ± 1.21) (2.62)	
Trigylicerides (mg/dl)	180.1 ± 95.1 (168)	150.1 ± 60.9 (140.5)	0.231
(mmol/l)	(2.04 ± 1.07) (1.90)	(1.70 ± 0.69) (1.59)	
25-hydroxy Vitamin D (ng/ml)	19.9 ± 6.1 (19.5)	26 ± 8.2 (26.4)	< 0.001
Calcium (mg/dl)	9.2 ± 0.5 (9.1)	9.4 ± 0.4 (9.4)	0.034
Magnesium (mg/dl)	2.1 ± 0.3 (2)	2.1 ± 0.4 (2.1)	0.086
Albumin (g/dl)	4 ± 0.4 (4.1)	4.2 ± 0.3 (4.1)	0.163
Potassium (mmol/l)	4.1 ± 0.5 (4.1)	4.3 ± 0.3 (4.3)	< 0.001
Uric acid (mg/dl)	6.4 ± 1.5 (6.19)	5.5 ± 1.2 (5.4)	0.004
Left atrium (mm)	41.2 ± 4.3 (41)	37.8 ± 3.9 (38)	< 0.001
Ejection fraction (%)	51.3 ± 9.1 (55)	55.2 ± 6.7 (55)	0.043

POAF, postoperative atrial fibrillation.

Univariate analysis identified age, DM, history of transcient ischaemic attack/stroke, COPD, heart failure, left atrial diameter, EF, and urea, creatinine, uric acid, potassium, calcium and 25-(OH) Vitamin D levels as significant factors for the development of POAF. Multivariate regression models revealed that COPD, DM, HF and left atrial diameter increased the probability of POAF independent of confounding factors (OR: 28.737, 95% CI: 0.836–16.118, p < 0.001 for COPD; OR: 11.486, 95% CI: 0.734–11.060, p = 0.001 for DM; OR: 15.430, 95% CI: 0.989–7.649, p = 0.006 for HF; OR: 1.245, 95% CI: 0.086–6.431, p = 0.011 for left atrial diameter).

## Discussion

AF is a growing global health concern and is linked to a wide range of medical complications, including heart failure, ischaemic stroke and death. It is estimated that AF may account for 10 to 15% of all strokes, with an associated increased mortality rate of up to 1.9-fold higher than without AF.^5^

COPD, HF, DM and left atrial diameter were found to be independent variables predicting the development of POAF. In previous studies, advanced age, male gender, chronic heart failure, pre-operative AF attacks, COPD, chronic renal disease, DM and the metabolic syndrome were reported to be pre-operative clinical parameters predicting the development of POAF.^6^

COPD is an independent risk factor for arrhythmias, especially AF and cardiovascular morbidity and mortality.^7^ COPD was found to be an important variable predicting the development of postoperative AF in this study. We believe that the relationship between COPD and POAF depends on hypoxia, hypercapnia, acidosis and inflammation.

AF is one of the most common co-morbidities in patients with HF, while HF is also common in AF patients. Previous studies reported that the prevalence of AF in patients with chronic HF ranged from 15 to 50%.^8^ HF was found to be an important variable predicting the development of postoperative AF in our study.

Aksakal and co-workers found DM increased the risk of developing AF.^9^ In our study, DM was found to be an important variable predicting the development of postoperative AF.

The Framingham Offspring study found that individuals with 25-(OH) Vitamin D < 37.5 nmol/l had a hazard ratio of 1.62 for the development of cardiovascular disease compared to those with a level of ≥ 37 nmol/l.10 Furthermore, Vitamin D insufficiency was associated with endothelial dysfunction and subclinical atherosclerosis.^11^ Another study pointed out that 25-(OH) Vitamin D levels were significantly lower in patients with coronary artery disease than in those without.^12^

VDRs are found in myocytes and fibroblasts in the heart.^13^ A number of animal studies have confirmed that VDRs play an important role in cardiac hypertrophy.^14^

The risk of new-onset AF is significantly higher with increased left atrial diameter and left atrial volume.^15^ In our study, left atrial diameter was found to be an important variable predicting the development of postoperative AF.

The role of Vitamin D deficiency in the onset of AF was suggested because of several potential mechanisms described previously.^16^ Vitamin D regulates inflammatory responses and up-regulates the expression of anti-inflammatory cytokines, such as IL-10, according to in vitro experiments.^17^ Also, Vitamin D regulates activity of the renin–angiotensin–aldosterone system (RAAS). Activated RAAS can lead to oxidative stress and inflammation, both of which could culminate in AF.^18^ It is assumed that tissue angiotensin II may induce apoptosis of the cardiomyocytes and contribute to changes in atrial structure.^19^

There were conflicting results regarding low 25-(OH) Vitamin D levels and AF. On one hand, several studies demonstrated a close association between Vitamin D deficiency and AF, such as Demir et al.,^20^ who found a strong relationship between Vitamin D deficiency and non-valvular AF. Chen and co-workers found that serum 25-(OH) Vitamin D level correlated with high-sensitivity C-reactive protein and left atrial diameter, and was significantly associated with AF in Chinese patients with non-valvular persistent AF.^21^ Hanafy et al.^22^ revealed the direct electromechanical effects on the left atrium after Vitamin D administration, and found that Vitamin D could effectively prevent or terminate AF.

On the other hand, no association was found between 25-(OH) Vitamin D levels and ischaemic heart disease, stroke or acute myocardial infarction, despite previous studies showing Vitamin D deficiency to be associated with increased incidence of these conditions.^23^-^25^ Rienstra et al.^26^ evaluated 2 930 participants of the Framingham Heart study during a follow-up period of 9.9 years and found no relationship between Vitamin D status and incident AF, concluding that Vitamin D deficiency does not promote the development of AF. Additionally, Qayyum et al.^27^ showed that there was no association between Vitamin D deficiency and type of AF or complications of AF. Another prospective cohort study based on the Rotterdam study did not support the hypothesis that Vitamin D level is associated with AF.^28^

Our study was the first to evaluate the predictive value of 25-(OH) Vitamin D level in the development of POAF. In recent studies, there has been a paradox between Vitamin D levels and AF, and a negative correlation between Vitamin D and left atrial diameter.^15^ In our study, although there was a significant negative correlation between Vitamin D and left atrial diameter, Vitamin D level was not an independent predictor for the development of POAF.

We believe that the paradoxical results between Vitamin D and AF could be related to the activation of the RAAS caused by Vitamin D insuffiency, increased levels of reactive oxygen radicals, and individual differences in receptor activity. Also, because of the negative correlation between Vitamin D level and left atial diameter, it could be hypothesised that Vitamin D insuffiency could lead to atrial dilatation, causing AF. Further randomised clinical studies are needed in this field.

Our study has some limitations. First, it was a retrospective study design. Second, AF was diagnosed by ECG monitoring in a hospital setting without performing a follow up after discharge. Third, the small sample size of this study was problematic. Fourth, measurement of Vitamin D levels occurred at a single point in time. Fifth, we did not determine parathyroid hormone levels.

## Conclusion

To the best or our knowledge, this study is the first to evaluate the relationship between POAF and 25-(OH) Vitamin D levels. Our study does not support the hypothesis that Vitamin D levels play a role in the aetiology of POAF. Further prospective, randomised studies with a larger number of patients are required to confirm our results.
